# Avenanthramides in Obesity-Related Metabolic Dysfunction: Mechanisms, Evidence Attribution, and Translational Priorities

**DOI:** 10.3390/nu18183066

**Published:** 2026-09-19

**Authors:** Xiaoyu Zhang, Hao Sun, Biaoxu Tao, Ziyang Li, Shuning Liu, Haojie Zhang, Chang Liu, Jiatong Wang

**Affiliations:** 1 School of Sports Training, Wuhan Sports University, Wuhan 430079, China; 2024410712@whsu.edu.cn (X.Z.); 2024410703@whsu.edu.cn (H.S.); 2024410681@whsu.edu.cn (B.T.); 2024410716@whsu.edu.cn (Z.L.); 2 School of Sports Science, Beijing Sport University, Beijing 100084, China; 2023013553@bsu.edu.cn; 3 Department of Sports Journalism and Management, Henan Sport University, Zhengzhou 450044, China

**Keywords:** avenanthramides, oats, obesity, metabolic inflammation, insulin resistance, hepatic steatosis, lipid metabolism, gut microbiota

## Abstract

Background: Obesity-related metabolic dysfunction includes adipose inflammation, hepatic steatosis, insulin resistance, dyslipidemia, and intestinal disturbances. Avenanthramides are oat-specific phenolic amides, and their contribution to metabolic responses to oat foods is difficult to attribute because beta-glucan and the wider oat matrix can affect the same endpoints. This narrative review examines their mechanisms, exposure biology, evidence attribution, and translational readiness. Methods: Cell, animal, pharmacokinetic, and human studies were organized by chemical definition and intervention format, including purified congeners, characterized foods, enriched matrices, and whole-oat products. Parent compounds, circulating metabolites, target tissues, and clinical outcomes were interpreted separately. Results: In cell and animal models, avenanthramides influence NF-κB-related inflammation, redox regulation, hepatic insulin signaling, glucose production, fatty-acid metabolism, and intestinal ecology. Human studies demonstrate dietary exposure to parent compounds and metabolites, while clinical evidence comes mainly from small enriched-food exercise studies. Among the studies identified and appraised in this narrative review, none established an avenanthramide-specific effect on adiposity, liver fat, insulin sensitivity, dyslipidemia, or other cardiometabolic outcomes. Conclusions: The current literature supports measurable exposure and candidate mechanisms; a validated anti-obesity effect requires targeted clinical trials. Next studies need stable, analytically defined preparations, metabolite-informed dose selection, matched food controls, and trials that link measured exposure with prespecified clinical outcomes.

## 1. Introduction

Obesity is not defined solely by excess body mass. Its clinical burden arises largely when adipose tissue can no longer accommodate a sustained energy surplus without inflammation, fibrosis, and ectopic lipid deposition [1,2]. Adipose insulin resistance increases the delivery of non-esterified fatty acids to the liver, while altered adipokine secretion and immune-cell recruitment increase systemic inflammation [1,2]. These disturbances contribute to dyslipidemia, impaired glucose regulation, metabolic dysfunction-associated steatotic liver disease (MASLD), and cardiovascular disease [1,2]. Weight reduction remains central to management, but dietary responses also depend on food structure, fiber, and co-occurring bioactive constituents [3,4]. The relevant question is whether the defined components of oat foods alter particular metabolic phenotypes.

Oats are widely used in dietary strategies for cardiometabolic health. Much of the established evidence is attributable to beta-glucan, a soluble fiber whose viscosity changes intestinal nutrient handling, postprandial glycemia, and cholesterol metabolism [3,4]. Oats also contain lipids, proteins, tocopherols, phenolic acids, and the oat-specific nitrogen-containing phenolic amides known as avenanthramides [5,6,7]. Avenanthramides raise a separate question: whether responses to oat foods, particularly in inflammation and redox signaling, involve constituents not shared by most cereals.

Avenanthramides were described as substituted *N*-cinnamoyl anthranilate alkaloids in oat groats and hulls, and more than 40 congeners have since been reported [5,6]. The three forms studied most often are avenanthramide A, B, and C, also designated 2p, 2f, and 2c according to the anthranilate–hydroxycinnamate nomenclature [5,8,9]. They differ in the hydroxycinnamic acid moiety and therefore in redox behavior, metabolism, and possibly biological activity [5,10]. Their concentrations vary substantially across cultivars and oat products, and the amount consumed in a serving of oats is considerably lower than the doses used in many cell and animal experiments [7,8,11]. Consequently, findings obtained with a purified compound, a concentrated extract, and an oat food cannot be treated as equivalent evidence.

Earlier work on avenanthramides focused mainly on inflammatory signaling and vascular cell responses [12,13]. These studies established biological plausibility and provided the foundation for obesity-related metabolic research. More recent investigations have extended the field: avenanthramides A, B, and C modified insulin signaling, gluconeogenesis, and glycogen synthesis in a fatty-acid-induced HepG2 model, whereas avenanthramide B and avenanthramide-rich extracts reduced weight gain and altered lipid or intestinal microbial outcomes in high-fat-diet-fed mice [14,15,16]. These findings are relevant to hepatic insulin resistance and lipid accumulation, but cell concentrations and animal dosing do not establish that ordinary oat intake produces the same effect in humans.

Human studies require interpretation by intervention format. Acute pharmacokinetic studies detect avenanthramides in plasma after concentrated extracts, oat cookies, oat bran, and commercial oat products [17,18,19]. Parent compounds are accompanied by methylated, glucuronidated, sulfated, and microbially reduced metabolites, some of which appear later than the initial postprandial peak [20,21]. Product physical form and subsequent host–microbial metabolism shape the measured exposure profile [22,23,24]. These observations matter because a cell exposed to a parent compound at a micromolar concentration does not reproduce the mixture, concentration, or timing of species measured after food consumption.

Existing reviews have addressed avenanthramide nomenclature, biosynthesis, antioxidant properties, and broad health effects [5,6,25]. The obesity-related literature is more scattered: relevant data come from adipose, hepatic, intestinal, vascular, pharmacokinetic, and food-intervention studies. A useful synthesis keeps those evidence streams separate and assigns effects according to the tested exposure.

This narrative review follows avenanthramides from chemical diversity, dietary occurrence, and processing stability through absorption and metabolism to their reported relevance in obesity-related metabolic dysfunction. It focuses on the gut, adipose tissue, liver, and downstream vascular consequences. Findings from purified congeners, chemically characterized extracts, avenanthramide-enriched foods, and whole-oat products are kept separate wherever the available evidence allows. The primary readers are nutrition and metabolism researchers, clinicians, and dietitians who need to interpret mechanistic and human evidence; food-development researchers may also use the exposure and comparator criteria when designing interventions.

## 2. Materials and Methods

PubMed/MEDLINE was used as the primary biomedical database. Records were also checked through Crossref and publisher pages, Google Scholar and targeted web-title searches, ClinicalTrials.gov, and backward citation chaining from cited studies and recent reviews. This broader source-checking was particularly important for food-chemistry, analytical-method, and trial-registration records that may not be indexed consistently in biomedical databases. The initial PubMed search covered database inception through 7 July 2026. Evidence extraction was completed through 31 July 2026, followed by a final PubMed refresh on 7 August 2026.

Search terms combined avenanthramide and oat terminology with terms for absorption, bioavailability, metabolism, microbiota, inflammation, oxidative stress, obesity, adipose tissue, insulin and glucose metabolism, lipid metabolism, hepatic steatosis, clinical trials, and interventions. Supplementary Materials provide the recorded PubMed query, narrative scope and selection rationale and evidence-charting domains. Two reviewers (X.Z. and H.S.) screened and selected the literature, extracted study information, and checked the extracted data. Only English-language literature was searched. Evidence extraction was completed through 31 July 2026. The recorded PubMed terminology comprised avenanthramide*, avenanthramides A, B, and C, their 2p, 2f, and 2c designations, and oat, oats, and Avena sativa. Targeted revision checks also paired these terms with extract, flour, bran, cookie, whole-oat, and whole-grain food forms.

Reporting of this narrative review includes the information sources and search terms, narrative scope, evidence-charting domains, and synthesis approach in the main text and Supplementary Materials. Table S1 is a representative map of cited studies chosen to illustrate differences in intervention format, exposure measurement, model or population, and the inference supported by each design. Table S2 provides a focused appraisal of the three enriched-cookie trials.

During revision on 7 September 2026, targeted checks of recent avenanthramide records in Europe PMC, original reports, publisher tables, and ClinicalTrials.gov were used to verify intervention details, safety reporting, references, and registry status. The full report of the 2016 enriched-cookie trial was additionally checked on 9 September 2026.

Evidence was attributed according to the tested exposure. Purified congeners were interpreted as evidence for compound-specific biological activity; analytically characterized enriched foods were interpreted as nutritionally relevant but potentially co-exposed interventions; and whole-oat trials were interpreted as food-level evidence unless matched contrasts and exposure verification supported stronger attribution. Because the evidence base spans cell models, animal studies, pharmacokinetic studies, registered trials, small human interventions, and whole-food trials, findings were synthesized narratively. The narrative design integrates chemistry, processing, pharmacokinetics, mechanisms, and human interventions to address evidence attribution across study types. Their populations, exposures, comparators, and outcomes do not form a uniform set for a pooled treatment-effect estimate; narrower clinical questions remain suitable for future systematic review.

## 3. Structure, Content, and Stability of Avenanthramides

### 3.1. Structure and Nomenclature

Avenanthramides are amides formed between an anthranilic acid derivative and a hydroxycinnamic acid or related phenylpropanoid [5,9]. Collins originally used an alphabetical system to distinguish compounds isolated from oat groats and hulls [9]. A later nomenclature identifies the anthranilate moiety with an uppercase or numerical component and the hydroxycinnamate moiety with a lowercase letter [5]. Both systems remain in use, which is why the same major compounds are commonly described as avenanthramide A (2p), avenanthramide B (2f), and avenanthramide C (2c).

Avenanthramide A contains a *p*-coumaroyl moiety, avenanthramide B contains a feruloyl moiety, and avenanthramide C contains a caffeoyl moiety [5,9]. Differences in hydroxylation and methoxylation influence electron donation, radical stabilization, and the substrates available for host and microbial metabolism [5,10]. Chemical antioxidant rank orders do not predict in vivo metabolic efficacy; absorption, conjugation, tissue exposure, and interaction with dietary components determine in vivo relevance alongside parent-compound reactivity in solution.

Oats contain less abundant avenanthramides in addition to A, B, and C [4,5]. High-resolution mass-spectrometric characterization has expanded the measurable avenanthramide profile beyond A, B, and C and showed that germination changes both total abundance and relative congener composition [26,27]. Reporting only total avenanthramides can therefore conceal meaningful differences in composition. Two extracts with the same total concentration may contain different proportions of A, B, C, and minor congeners, whereas a purified-compound experiment addresses a much narrower chemical exposure.

The terminology used to describe avenanthramides also requires care. They are often called polyphenols because they possess phenolic groups and are discussed alongside cereal phenolics, but chemically they are phenolic amides or phenolic alkaloids [5,6]. The amide linkage can be retained, reduced, hydrolyzed, or modified after ingestion, generating metabolites with properties that differ from those of the original food compound [20,21,23]. For that reason, this review treats avenanthramides as phenolic amides rather than as interchangeable members of a general oat-phenolic pool.

### 3.2. Content and Distribution in Oat Foods

Avenanthramides occur throughout the oat kernel but are concentrated in outer tissues, which helps explain their presence in bran-rich fractions and whole-grain products [7,8]. Surveys of commercial foods report wide variation, from low concentrations in some breads and highly processed products to higher concentrations in oat bran, flakes, groats, and selected cultivars [7,8]. Independent analyses of Finnish cultivars and Canadian genotype-by-environment panels likewise found marked variation in avenanthramides and accompanying phenolic acids [28,29]. Actual intake from an oatmeal consequently depends on the analyzed edible product, milling fraction, formulation, and serving size. Product labels rarely declare avenanthramide content, so the same food category can provide substantially different exposure.

Avenanthramide content varies substantially by genotype. Comparative analyses of oat cultivars have reported several-fold differences in total concentration and in the relative abundance of the major congeners [11]. Avenanthramide content is a variable product property. Concentrations should specify product type, assay, congener panel, and dry-matter or as-consumed basis; values from different methods should not be pooled [7,8,27]. Authentic-standard LC-MS/MS work published in 2026 demonstrates congener-dependent calibration bias in commercial oat products, with implications for administered-dose accuracy [30].

### 3.3. Processing and Stability

Commercial oats are commonly stabilized by steaming or other heat treatments [31,32]. Processing can change avenanthramide concentration through thermal degradation, release from the matrix, moisture-dependent reactions, or redistribution among milling fractions [31]. The direction and magnitude of change should not be generalized across products because temperature, time, moisture, and subsequent storage differ among processes. Germination can increase content and change congener composition, depending on the conditions; enriched products require analysis of AVNs and accompanying food constituents [26,33,34]. Cooking, steaming, baking, extrusion, fermentation, and germination can alter measured total avenanthramides or relative congener abundance through degradation, release, redistribution, and changes in extractability.

Milling changes the proportion of outer kernel tissues, and bran-rich fractions tend to retain more avenanthramides than refined oat ingredients [7,8]. Extrusion, baking, fermentation, and extraction add further variability; apparent losses can reflect chemical degradation, whereas apparent increases can reflect improved extractability after matrix disruption [31,35,36]. Analytical methods also differ in solvent, hydrolysis, standards, and whether free or conjugated forms are measured; dedicated studies demonstrate that extraction conditions alter the minor-polar profile and measured avenanthramide recovery [35,36]. Cross-study comparisons need to account for the analytical method as well as the food matrix.

Storage is another source of variation. Phenolic profiles can change with time, temperature, oxygen exposure, and residual enzyme activity [32]. A product standardized at manufacture may therefore not deliver the same quantity at the end of its shelf life unless stability has been established. Clinical trials should report analyzed avenanthramide content in the batches participants consume rather than relying on development-stage values or food-composition estimates. The effects of refrigeration and light exposure vary across edible oat products and should be evaluated with documented storage conditions. Intervention studies should document storage conditions and analyze the batches consumed at relevant time points.

The food matrix influences more than chemical stability. Avenanthramides coexist with starch, protein, lipids, beta-glucan, and other phenolics, and interactions with starch can change both avenanthramide bioaccessibility and starch digestibility in vitro [37]. The viscosity and physical structure of oat products can also modify gastric emptying, intestinal release, and absorption timing [22,38]. A 2025 pharmacokinetic study reported different time-to-peak values for several avenanthramides after solid and liquid oat products, even though both formats produced measurable plasma exposure [22]. The dose expressed in milligrams therefore does not fully define a nutritional intervention.

Processing and matrix effects define the boundary for metabolic interpretation. Whole-oat interventions provide food-level evidence because they contain multiple active components [7,8,37]. Purified or concentrated preparations strengthen mechanistic attribution while representing distinct absorption kinetics, co-exposures, and gastrointestinal effects [18,19,21]. Interpretation should follow the intervention that was tested. Table 1 summarizes the chemical and food-processing information needed to interpret exposure and design obesity-related intervention studies. A standardized extract defines one preparation; translation to conventional foods requires comparable congener composition, dose, matrix, and measured systemic exposure.

## 4. Digestion, Absorption, Metabolism, and Tissue Exposure

### 4.1. Intestinal Absorption and Phase II Metabolism

The biological relevance of an avenanthramide mechanism depends on whether a parent compound or metabolite reaches the proposed site at an appropriate concentration. Human pharmacokinetic studies have detected absorbed avenanthramides, but the pattern is characterized by low plasma concentrations, rapid conjugation, and variation among compounds and individuals [18,19,22]. In an early crossover study using an avenanthramide-enriched extract, avenanthramides A, B, and C appeared in plasma as free and conjugated forms, with maximum concentrations in the nanomolar range and a dose-related increase after the larger extract dose [18]. This established bioavailability for a concentrated preparation, which represents a different exposure from a normal serving of oats.

Subsequent studies used oat foods. After consumption of oat cookies made with avenanthramide-containing flour, parent compounds appeared in plasma during the postprandial period and were eliminated over several hours [19]. Oat-bran studies detected parent avenanthramides, phenolic acids, and numerous metabolites in plasma and urine [21]. These experiments are closer to dietary exposure; polarity, substitution, and metabolism produce distinct concentration-time profiles across compounds [19,21,22].

Cellular transport work using a human colon adenocarcinoma model suggests that avenanthramides 2p, 2f, and 2c can cross the intestinal epithelium through more than one route, with compound-specific absorption rates [39]. As with any Caco-2-type model, transporter expression, barrier properties, and the absence of digestion and microbiota limit direct extrapolation. Its main value is to show that structure affects intestinal handling and that passive diffusion alone is unlikely to explain every congener.

Once absorbed, avenanthramides undergo extensive phase II metabolism. Methylated metabolites have been identified in human plasma, and glucuronidated and sulfated forms contribute to circulating and urinary profiles [20,21]. Conjugation lowers the concentration of free parent compound and may change cellular uptake and reactivity. Cell experiments limited to unconjugated parent compounds are best read as mechanism screens unless circulating metabolites are tested as well.

Pharmacokinetic modeling of solid and liquid oat consumption showed that food form affects absorption and that measured avenanthramides have relatively short elimination half-lives [22]. The study evaluated biomarkers of oat intake rather than metabolic efficacy. For intervention design, its value lies in modeling repeated exposure and in showing why transient plasma concentrations and matrix-dependent kinetics matter when selecting doses and sampling times.

### 4.2. Microbial Biotransformation and Interindividual Variation

Not all ingested avenanthramides are absorbed in the upper gastrointestinal tract. Compounds reaching the colon can be transformed by the microbiota, creating a phase of exposure later than the initial postprandial appearance of parent molecules. Experimental work with avenanthramide C showed side-chain reduction and other transformations in mice and human fecal microbiota, with some resulting metabolites retaining activity in the assays used [23]. These data broaden the relevant exposure from the parent compound alone to both food-derived and microbiota-derived molecules.

A controlled whole-grain oat study in healthy adults demonstrated distinct avenanthramide metabotypes. Parent avenanthramides reached earlier plasma maxima, whereas dihydro-metabolites appeared later, consistent with colonic microbial conversion [24]. Faecalibacterium prausnitzii abundance was associated with reduced-metabolite generation in that study [24]. This association identifies microbial composition as a potential source of variation in systemic exposure.

Whether obesity alters avenanthramide metabolite profiles requires direct study in people with obesity. Available pharmacokinetic data indicate that measuring parent compounds alone can miss conjugated and microbially reduced exposure, whereas fecal community composition alone cannot establish which metabolites entered the circulation [18,19,22].

Future intervention studies should integrate product analysis, repeated plasma or urine measurements, targeted assays of major conjugated and reduced metabolites, and a prespecified microbiome component [18,19,22]. A focused panel of known human metabolites can show whether the tested food delivered its intended exposure and whether response varied by metabotype. This design follows current recommendations for nutrition feeding trials [40].

### 4.3. Tissue Distribution and Biological Relevance of Metabolites

Direct evidence of avenanthramide tissue distribution in humans is not available. In rats, orally administered avenanthramides were detected in liver, heart, and skeletal muscle, indicating that under gavage conditions the measured forms are not confined to plasma [41]. This supports biological plausibility for hepatic and vascular action, while the therapeutic tissue concentration achieved by a dietary serving remains unmeasured. Available hepatic studies do not isolate the parent compound from host- or microbiota-derived metabolites as the causal species. The HepG2 experiment applied parent avenanthramides A, B, and C directly [14], while mouse studies tested avenanthramide B or an enriched extract without metabolite-blocking, rescue, or tissue-speciation experiments [15,16]. Microbial metabolites were measured in experimental biotransformation and human metabotype studies [23,24], but their mediation of hepatic outcomes has not been tested.

The gap between experimental and nutritional exposure is apparent in hepatic insulin-resistance work. Avenanthramides A, B, and C improved glucose handling and insulin-related signaling in HepG2 cells at 100 μmol/L [14], whereas postprandial concentrations after oat foods or enriched preparations are generally in the nanomolar range [18,19,22]. Protein binding, tissue accumulation, repeated exposure, and active metabolites could affect the comparison, but they do not erase this multi-order-of-magnitude difference. Mechanistic studies need to justify their concentrations against human pharmacokinetics.

**Figure 1:**
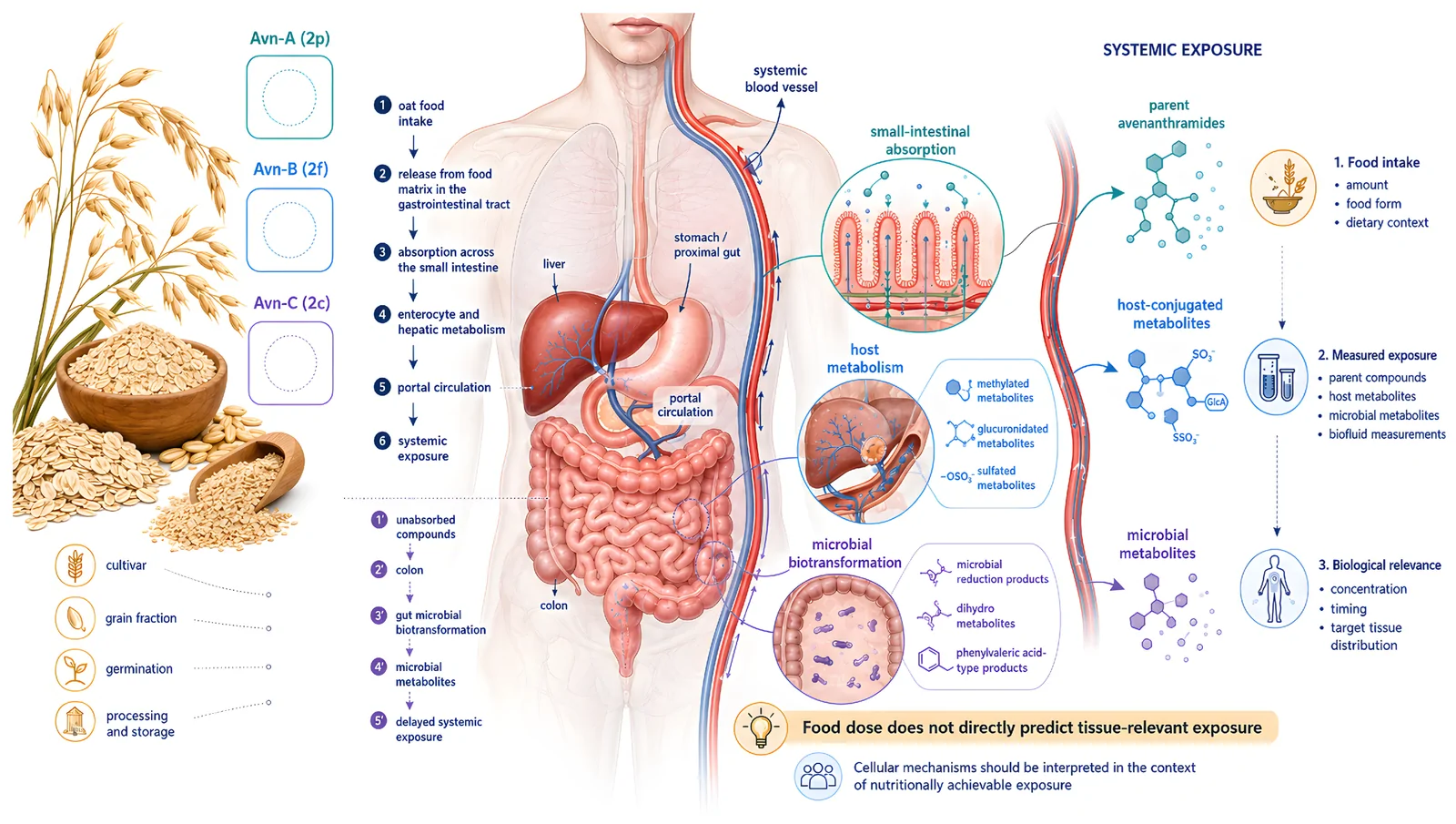
From oat food to tissue-relevant avenanthramide exposure in humans. Food-matrix determinants influence release, small-intestinal absorption, phase II host metabolism, colonic microbial biotransformation, and the timing and identity of detectable analytes. Tissue-relevant exposure requires targeted measurement of parent, host-conjugated, and microbial metabolites in relevant biofluids because the administered food dose alone is insufficient [18,19,22].

For the following sections, findings are classified by the chemical definition of the intervention and the available exposure evidence. Purified parent compounds establish compound-specific biology but not necessarily nutritionally achievable exposure. Characterized enriched foods retain greater dietary realism but also retain co-exposures. Whole-oat studies test the food while providing the weakest attribution to a single component [15,42,43]. Figure 1 summarizes the exposure sequence and the measurements needed to interpret it.

## 5. Mechanisms Relevant to Obesity-Related Metabolic Complications

Mechanistic studies of avenanthramides address modulation of obesity-related metabolic dysfunction; weight-loss efficacy requires separate clinical trials. Obesity exposes adipose tissue, liver, intestine, vasculature, and immune cells to nutrient excess, inflammatory signaling, redox imbalance, ectopic lipid deposition, and impaired insulin action [1,2]. Avenanthramides have been tested at several nodes in this network, but the evidence varies by compound, model, dose, and intervention format [7,15,18]. The sections below organize the findings by tissue while retaining those distinctions.

### 5.1. Oxidative Stress and Inflammatory Signaling

Low-grade inflammation links expanded adiposity to insulin resistance, endothelial dysfunction, hepatic steatosis, and cardiometabolic disease [1,2]. NF-κB is a transcriptional hub through which cytokines, lipotoxic stress, oxidative stimuli, and innate immune activation can worsen tissue dysfunction. Avenanthramides entered metabolic research partly because endothelial studies reported reduced cytokine expression and NF-κB-dependent signaling after avenanthramide exposure [12].

The strongest compound-specific evidence concerns anti-inflammatory and antioxidant responses in cell systems. In endothelial cells, avenanthramides reduced IL-1β- or TNF-α-stimulated inflammatory mediator expression and NF-κB activation [12]. Similar pathways are relevant to obesity biology, but the experiments used parent compounds at concentrations above typical postprandial plasma levels after oat foods [18,19,22]. They support inflammatory signaling as a candidate mechanism; whether normal oat intake suppresses systemic inflammation through this pathway requires human testing.

Redox findings are difficult to generalize across models. Avenanthramides differ in hydroxylation and methoxylation, and antioxidant ranking in chemical assays does not necessarily predict in vivo efficacy [10]. More informative studies measure cellular stress signaling, antioxidant enzyme activity, lipid peroxidation, or inflammatory outputs in relevant models. The exposure used in these experiments should still be compared with pharmacokinetic data because circulating species include methylated, glucuronidated, sulfated, and microbially reduced metabolites [20,21,22].

Inflammation is a plausible cross-cutting mechanism for avenanthramides. Human evidence comes from small, exercise-based enriched-food studies rather than obesity-focused efficacy trials [43,44,45]. At present, cellular and animal findings justify metabolite-informed human tests of inflammatory and redox hypotheses; clinical anti-inflammatory claims require such trials.

### 5.2. Adipose Tissue Dysfunction and Systemic Inflammation

Adipose tissue is an organizing tissue in obesity-related metabolic dysfunction. During sustained positive energy balance, adipocyte hypertrophy, local hypoxia, extracellular matrix remodeling, macrophage recruitment, and altered adipokine secretion can increase systemic inflammatory and lipolytic stress [1,2]. This promotes non-esterified fatty acid flux to the liver and contributes to whole-body insulin resistance [1,2]. Avenanthramide effects on adipose tissue have been studied less directly than endothelial, hepatic, or intestinal effects.

Direct evidence that purified avenanthramides remodel adipose tissue inflammation is thinner than the evidence for endothelial, hepatic, or intestinal effects. Avenanthramide B and avenanthramide-rich extracts have reduced weight gain, improved lipid indices, or altered inflammatory and microbial outcomes in high-fat-diet mouse models [15,16]. These studies leave adipose-tissue anti-inflammatory action unresolved, particularly when adipose histology, adipokines, or macrophage markers were not measured.

Current data link avenanthramides to adipose-tissue biology indirectly. Effects on inflammatory signaling, intestinal exposure, or hepatic lipid metabolism could secondarily reduce adipose stress, but this remains an inference [12,15,16]. Future studies should measure adipocyte size distribution, crown-like structures, macrophage polarization, adiponectin and leptin, lipolysis-related enzymes, and insulin-stimulated glucose uptake. Adipose tissue therefore remains a proposed target pending direct measurement.

### 5.3. Hepatic Glucose Metabolism and Insulin Resistance

The liver is a relevant target because obesity-related insulin resistance is linked to excessive hepatic glucose production, impaired glycogen synthesis, de novo lipogenesis, and ectopic lipid accumulation [1,2]. In a free-fatty-acid-induced insulin-resistance HepG2 model, avenanthramides A, B, and C improved glucose handling and modulated proteins involved in insulin signaling, glycogen synthesis, and gluconeogenesis, including IRS-1, PI3K, Akt, GSK3β, FoxO1, AMPK, G6Pase, and PEPCK [14].

The HepG2 study identifies hepatic metabolic nodes that warrant translation [14]. The next studies should pair measured avenanthramide exposure with fasting insulin, hepatic insulin sensitivity, postprandial glucose handling, or liver-derived biomarkers.

The key translational issue is concentration. The HepG2 study used 100 μmol/L avenanthramides, whereas human postprandial concentrations after oat products are generally reported in the nanomolar range [14,18,22]. HepG2 cells also lack the multicellular architecture of liver tissue, immune-cell crosstalk, bile-acid signaling, and intestinal input. These data support hepatic glucose metabolism as a mechanistic hypothesis; clinical claims require exposure-matched human studies.

### 5.4. Lipid Metabolism, Hepatic Steatosis, and Dyslipidemia

Among obesity-related endpoints, lipid metabolism currently has the strongest animal evidence for a direct avenanthramide intervention. In high-fat-diet-fed mice, avenanthramide B reduced weight gain and improved hepatic and serum lipid indices, with transcriptomic and biochemical signals implicating fatty-acid metabolism [15]. An earlier high-fat-diet study using an avenanthramide extract similarly reported lower weight gain, oxidative stress, inflammatory markers, and microbial shifts but has weaker compound attribution because the intervention was an extract [16].

The avenanthramide B model uses a defined congener and links phenotypic outcomes with hepatic gene-expression patterns and microbial remodeling [15]. The dose may not correspond to ordinary dietary exposure, the effects were demonstrated in mice receiving a high-fat diet, and direct hepatic action remains difficult to separate from gut-mediated mechanisms. Reduced weight gain in this model supports a preclinical anti-obesity signal; human translation requires exposure-matched trials.

Intervention type determines the strength of attribution. Purified avenanthramide B provides compound-specific animal evidence for lipid metabolism regulation [15]. Avenanthramide-rich extracts provide food-relevant but less compound-specific evidence [16]. Whole-oat interventions can improve metabolic outcomes, but the effects may reflect beta-glucan, food structure, or multiple coexisting constituents unless avenanthramide exposure is measured [3,42,46].

### 5.5. Intestinal Lipid Handling, Barrier Function, and Gut Microbiota

The intestine is both an exposure site and a metabolic target. Avenanthramides are released from the oat matrix, absorbed to different extents, conjugated by host tissues, and transformed by colonic microbiota [22,23,24]. Intestinal lipid absorption, bile-acid signaling, microbial metabolites, barrier integrity, and low-grade endotoxemia can plausibly connect oat-derived compounds with hepatic, adipose, and systemic metabolic outcomes. This makes the gut a testable bridge for linking exposure to downstream metabolic outcomes.

The most distinctive evidence concerns microbial biotransformation and metabotypes. Avenanthramide C can be reduced by mouse and human microbiota to dihydro-metabolites, and whole-grain oat intake has been associated with interindividual differences in avenanthramide metabolite profiles [23,24]. The association between Faecalibacterium prausnitzii and reduced-metabolite generation suggests that the same oat food can produce different circulating exposures in different individuals [24]. Whether these metabolite patterns predict metabolic response in obesity or MASLD remains unknown. A separate ovalbumin-food-allergy mouse model reported improved colonic barrier markers and altered microbial and short-chain-fatty-acid profiles after avenanthramide administration [47]. That experiment supports the intestine as a candidate mechanistic site; its allergic-injury model addresses intestinal injury rather than obesity treatment.

A randomized study in 68 adults with metabolic syndrome illustrates both the food-to-host pathway and the attribution limit for avenanthramides. In two parallel dietary interventions, a two-day high-dose oat diet increased circulating ferulic acid and dihydroferulic acid and reduced LDL and total cholesterol relative to a macronutrient-adapted oat-free control. Replacing one habitual meal per day with 80 g of oats for six weeks altered the phenolic metabolome but did not reduce lipid markers between groups. Multi-omics integration linked plasma phenolic metabolites and microbial features to cholesterol response, and in vitro fermentation supported microbial generation of phenolic metabolites from oats [42].

This trial supports food-level attribution. Whole-oat interventions simultaneously deliver beta-glucan, dietary fiber, phenolic acids, minerals, and a food matrix that can change energy intake and microbial substrate availability [4,42]. The reported candidate mediators were ferulic acid, dihydroferulic acid, and related microbial phenols, whereas avenanthramides served principally as intake biomarkers [42]. The results suggest that microbial phenolic metabolism may contribute to food-level oat effects; identifying a causal phytochemical requires a matched exposure contrast.

Animal work with avenanthramide B and C points toward gut microbial and barrier-related mechanisms during high-fat feeding or experimental colitis [15,16,48]. These results generate hypotheses about obesity-related metabolism; causal attribution requires linked metabolite, barrier, and intervention tests. Evidence becomes stronger when microbial changes are linked to measured metabolites, barrier markers, lipid absorption, hepatic outcomes, and causal tests such as colonization or metabolite rescue.

Testing intestinal mediation requires three linked questions. Does the intervention deliver parent and metabolite exposure? Does exposure change intestinal lipid handling, barrier function, inflammatory tone, or microbial products? Do those intestinal changes explain downstream liver, adipose, or systemic outcomes? Most existing studies address only part of this sequence [23,24,42].

### 5.6. Vascular Dysfunction and Downstream Cardiometabolic Risk

Vascular dysfunction is not the central obesity phenotype, but it is a route through which obesity-related metabolic disease becomes clinically consequential [1,2]. Endothelial activation, oxidative stress, impaired nitric oxide bioavailability, smooth-muscle proliferation, and vascular inflammation contribute to hypertension, atherosclerosis, and cardiovascular events [1,2]. In vascular cell models, avenanthramide exposure inhibited inflammatory signaling or smooth-muscle proliferation and increased nitric oxide production [12,13].

Vascular-cell studies connect avenanthramides with downstream cardiometabolic mechanisms. Cell models identify candidate mechanisms such as NF-κB suppression, nitric oxide-related signaling, and inhibition of smooth-muscle proliferation [12,13]. Human translation would require changes in endothelial function, blood pressure, vascular inflammatory biomarkers, or oxidized lipid outcomes together with verified exposure.

**Figure 2:**
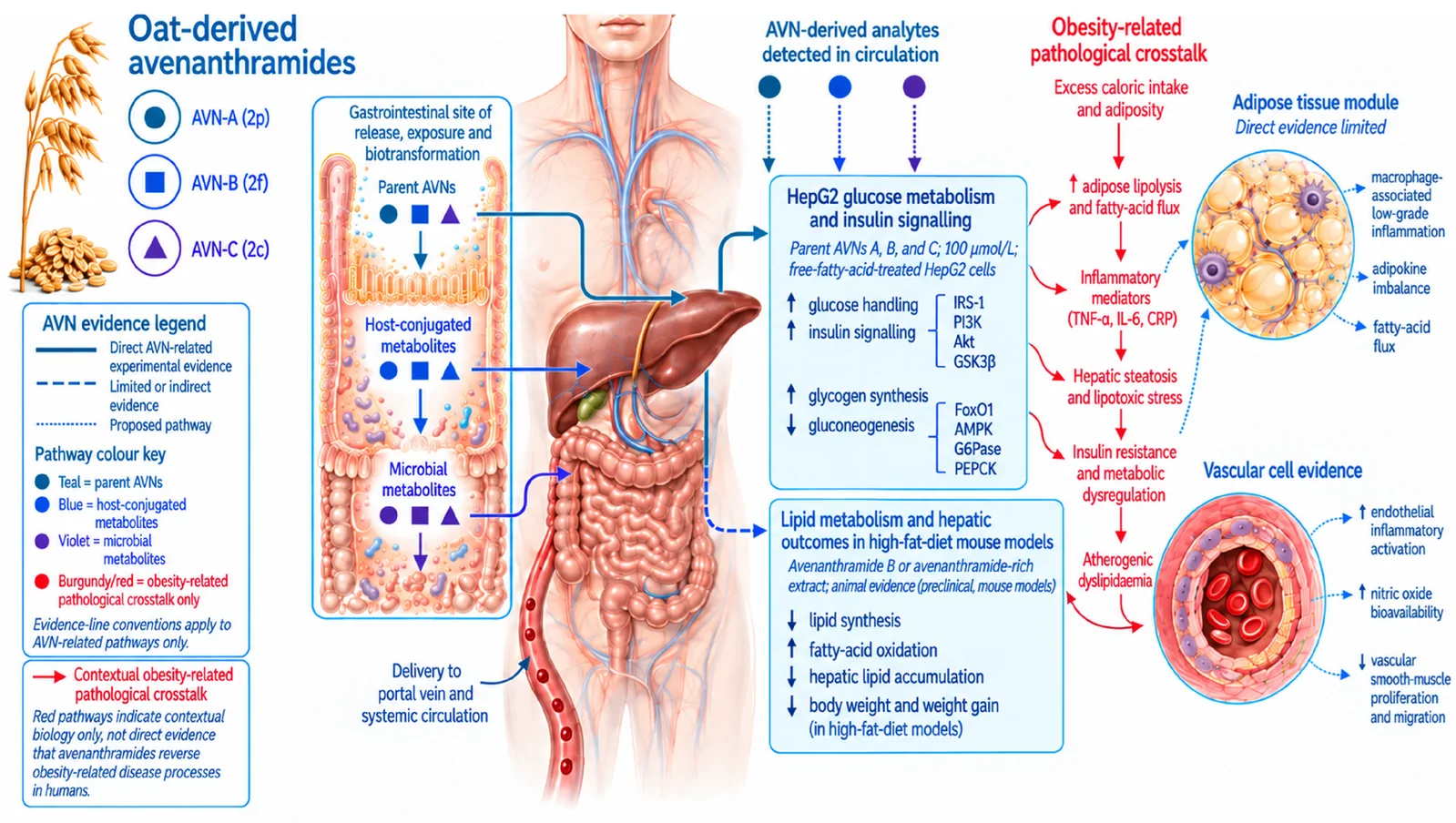
Evidence-aware working model for avenanthramides in obesity-related metabolic dysfunction. Solid, dashed, and dotted arrows denote direct, limited/indirect, and proposed AVN-related evidence, respectively; teal, blue, violet, and burgundy/red indicate parent AVNs, host-conjugated metabolites, microbial metabolites, and contextual obesity-related pathological crosstalk. Arrowheads indicate pathway direction. Red pathways are contextual only and do not imply reversal by AVNs in humans. The HepG2 module reflects parent AVNs A, B, and C at 100 μmol/L [14], whereas lipid, vascular, and microbial pathways summarize evidence from separate studies [12,13,14,15,16,23,24]. Abbreviations: AVN, avenanthramide; HepG2, human hepatocellular carcinoma cell line; IRS-1, insulin receptor substrate 1; PI3K, phosphoinositide 3-kinase; Akt, protein kinase B; GSK3β, glycogen synthase kinase 3 beta; FoxO1, forkhead box O1; AMPK, AMP-activated protein kinase; G6Pase, glucose-6-phosphatase; PEPCK, phosphoenolpyruvate carboxykinase; TNF-α, tumor necrosis factor alpha; IL-6, interleukin-6; CRP, C-reactive protein.

Vascular evidence extends the mechanistic rationale for avenanthramides beyond adiposity, particularly under oxidative and inflammatory stress [12,13]. Complementary vascular-cell work reported antiatherogenic actions of oat phenolic preparations and linked avenanthramide-C-mediated smooth-muscle growth inhibition to cell-cycle arrest [49,50]. These observations remain vascular-model evidence and should be interpreted separately from weight-loss activity and from hepatic or intestinal mechanisms; the experimental model, dose, and endpoint should remain explicit. Figure 2 summarizes the direct, limited or indirect, and proposed pathways.

## 6. Intervention Formats and Evidence Attribution

**Figure 3:**
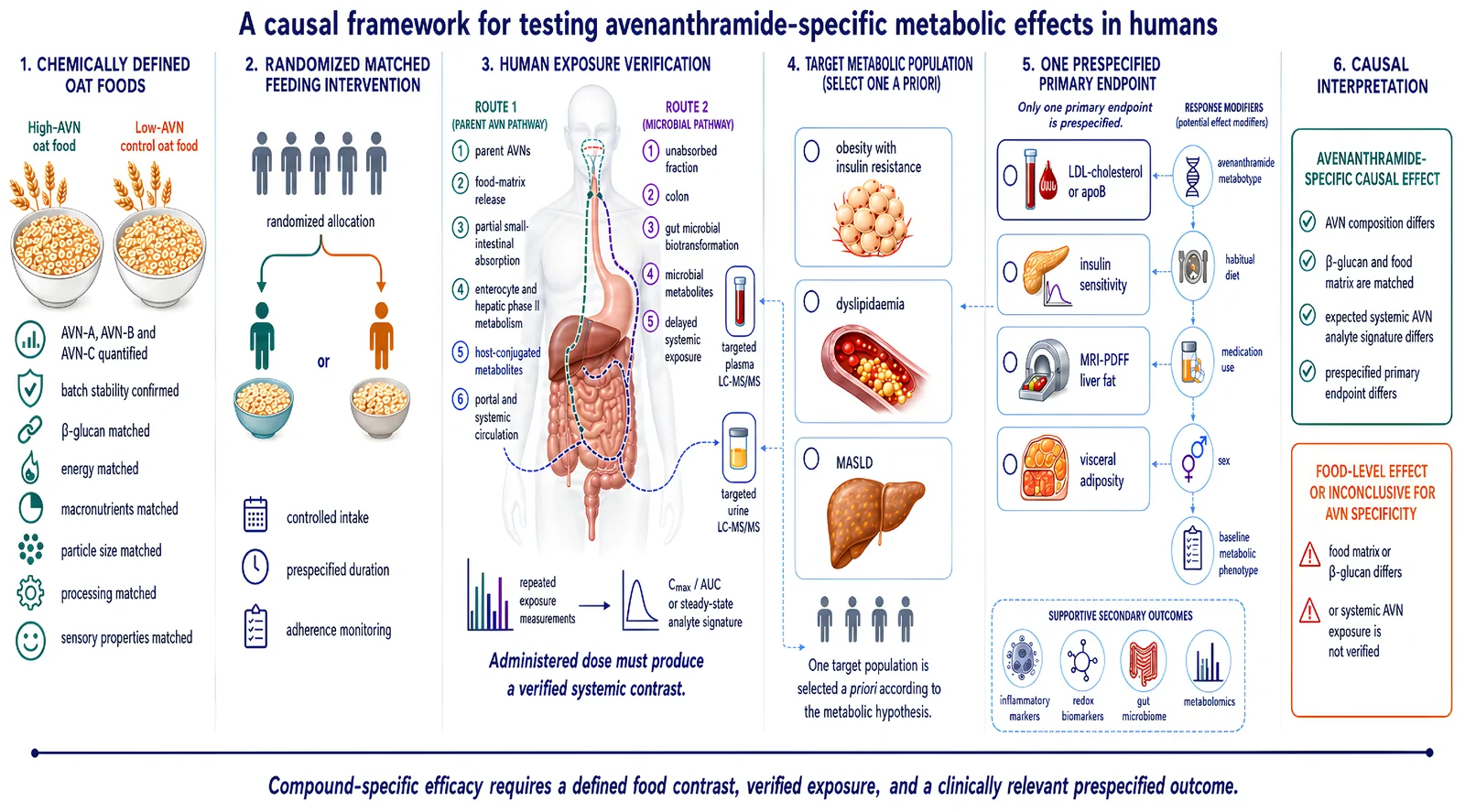
Framework for testing avenanthramide-specific metabolic effects in humans. Green and orange denote high- and low-AVN intervention arms; teal/blue and violet arrows indicate host and microbial exposure routes, respectively; dotted connectors indicate candidate effect modifiers. The bottom horizontal arrow summarizes progression from a chemically defined intervention to causal interpretation. Causal attribution requires matched food matrices, verified systemic exposure, a prespecified target population, and a prespecified primary endpoint [18,19,22]. Abbreviations: AVN, avenanthramide; LC-MS/MS, liquid chromatography–tandem mass spectrometry; Cmax, maximum concentration; AUC, area under the concentration–time curve; LDL, low-density lipoprotein; apoB, apolipoprotein B; MRI-PDFF, magnetic resonance imaging–proton density fat fraction; MASLD, metabolic dysfunction-associated steatotic liver disease.

Evidence should be interpreted according to the nutritional object that was tested, not solely according to the reported outcome (Figure 3). Purified congeners establish compound-specific biology at defined exposures; translation requires comparison with nutritionally attainable exposure. Enriched foods increase avenanthramide exposure within an edible matrix but retain co-exposures. Whole-oat foods test feasibility and clinical relevance with the least component-specific attribution [15,42,43]. This sequence links mechanistic findings to human interpretation.

### 6.1. Purified Congeners and Defined Preparations

Purified avenanthramides determine whether a particular congener can alter a biological process. The high-fat-diet mouse study of avenanthramide B is a useful in vivo example for obesity-related lipid metabolism because it combined a defined compound with phenotypic, biochemical, transcriptomic, and microbial outcomes [15]. Its translational scope is defined by the dose delivered and the lack of separation between direct hepatic action and secondary effects of altered intake and intestinal ecology.

Cell models narrow attribution further: they identify candidate insulin-signaling and gluconeogenic targets, while food efficacy requires evidence [14]. Translation requires evidence that moves from purified parent compounds to measured circulating metabolites, analytically characterized foods, and trials with clinically meaningful outcomes [18,19,21].

### 6.2. Avenanthramide-Enriched Foods and Functional Matrices

Avenanthramide-enriched foods retain a defined contrast while remaining closer to ordinary eating than purified compounds. In three small double-blind trials, participants consumed oat cookies differing in avenanthramide content for eight weeks, with standardized eccentric-exercise challenges before and after supplementation [43,44,45]. These trials assessed exercise-associated inflammatory and oxidative responses; obesity treatment requires a different clinical design. The food contrast also requires verification of beta-glucan, other phenolics, and systemic AVN exposure. Study-specific designs and reporting limits are appraised in Section 7.2 and Table S2.

Even when the avenanthramide contrast is explicit, product differences can remain. Heating, flour source, macronutrients, other phenolics, and dietary displacement may still differ between foods [7,31,37]. Future enriched-food trials should report each major congener, intervention stability, beta-glucan content, comparator composition, and postprandial parent and metabolite exposure [40].

### 6.3. Whole-Oat Foods and Food-Level Effectiveness

Whole-oat trials answer whether an oat food improves a clinically relevant phenotype under realistic conditions. A small 12-week trial in adults with obesity reported improvements in anthropometric, lipid, and liver-function measures after a beta-glucan-containing oat cereal [46]. Whole-grain oats also improved insulin sensitivity, plasma cholesterol, and gut microbiota composition in a mouse model [51]. These findings support food-level investigation; neither study measured the isolated contribution of avenanthramides. A systematic review separating whole-oat and isolated beta-glucan interventions reinforces the need to keep food-level and compound-level claims distinct [52].

The metabolic-syndrome randomized trial also demonstrates this attribution boundary. Its strengths include dietary control and objective phenolic and microbiome measurements within a prespecified multi-omics framework [42]. Measuring the biological species that reach circulation makes food trials more informative; avenanthramide-specific efficacy still requires a direct clinical trial.

## 7. Human Evidence and Clinical Interpretation

### 7.1. Exposure and Biomarker Evidence

After enriched mixtures and oat foods, human pharmacokinetic studies have detected avenanthramides and several conjugated or microbially derived metabolites [18,19,22]. These measurements establish exposure; efficacy requires clinical endpoints. In future trials, their immediate use is to document adherence and analyte exposure before clinical benefit is assessed.

### 7.2. Inflammation and Oxidative-Stress Outcomes

The direct human evidence for avenanthramide-related inflammatory effects comes primarily from controlled exercise studies. In a double-blind trial, 16 postmenopausal women consumed cookies providing 9.2 or 0.4 mg avenanthramides per day for eight weeks (8 per arm) [43]. Participants were recruited in Madison, Wisconsin; mean baseline body mass index (BMI) was 29.01 versus 26.40 kg/m^2^. The investigators described IL-1β as nearly 50% lower with the higher dose at rest and 24 h after the post-intervention downhill-walking challenge, with no difference at 48 h. No numerical between-group difference with a 95% confidence interval was reported for this contrast. Body weight and BMI were unchanged, creatine kinase (CK) did not differ by group, and total antioxidant capacity increased in both groups. Protocol and sampling details are given in Table S2 [43].

A similarly sized trial in 16 young women (8 per arm) compared the same cookie doses for eight weeks and reported lower exercise-associated IL-6 and NF-κB activity with the higher dose [44]. Participants were recruited in Madison, Wisconsin, were aged 18–30 years, and had a mean baseline BMI of 23.9 versus 23.3 kg/m^2^. They completed downhill-running challenges before and after supplementation (Table S2). Table 2 reports a post-intervention resting reduced glutathione (GSH) of 5.88 ± 0.08 versus 5.38 ± 0.09 μmol/L (mean ± SEM; between-group *p* < 0.05), compared with baseline values of 5.53 ± 0.09 versus 5.52 ± 0.09 μmol/L. That table provides no treatment-difference confidence interval. CK and TNF-α responses improved in both arms. The full report presents inflammatory outcomes as mean ± SEM graphs and *p* values, without numerical between-group effect estimates or 95% confidence intervals for IL-6, NF-κB, or neutrophil respiratory burst [44].

A later trial included 24 young, non-obese adults (12 per arm) and compared two cookies providing 20.6 mg/day AVNs with non-detectable AVNs, reported as 0 mg/day, for eight weeks [45]. Participants were recruited from Minneapolis–Saint Paul; mean age was 23.0 years and BMI 22.3 kg/m^2^. An eight-week washout separated the initial exercise test from supplementation. CK was 19.5% and 23.6% lower at 24 and 48 h POST versus PRE across allocations; CK and pain improvements were not AVN-specific. Some inflammatory biomarkers showed treatment-related interactions, but the IL-6 interaction was not conventionally significant (*p* = 0.082). The full report gives mean ± SEM graphs and *p* values, without numerical treatment-effect estimates and 95% confidence intervals for those interaction contrasts. Exercise and sampling protocols and the individual interactions are summarized in Table S2 [45].

Across these trials, the findings support a candidate effect on acute inflammatory responses to muscle-damaging exercise [43,44,45]. Obesity, MASLD, dyslipidemia, and type 2 diabetes require studies with disease-relevant endpoints. A systematic review of 23 oat-related randomized trials found no overall change in the inflammatory markers assessed, with only subgroup signals for C-reactive protein and interleukin-6 [53]. Clinical translation requires metabolically at-risk populations, prespecified endpoints, and quantified parent and metabolite exposure. Multiple correlated endpoints and sampling times, limited multiplicity reporting, and possible adaptation to repeated exercise further constrain interpretation. Common pre–post improvements should be separated from between-group effects; Table S2 details the available estimates and their uncertainty.

### 7.3. Weight, Lipids, Glycemia, and Liver Outcomes

The clinical evidence is stronger for oats as foods than for avenanthramides as an isolated active. A systematic review and meta-analysis of 74 randomized trials reported lower total and LDL cholesterol, with small improvements in anthropometric measures, after oat supplementation interventions; inflammatory and oxidative-stress evidence was scarce and inconsistent [3]. A second meta-analysis found lipid improvements after whole-oat and isolated beta-glucan interventions, showing that food-level evidence alone cannot identify avenanthramides as the causal constituent [52]. Two recent controlled food trials reinforce that distinction. In adults at risk of type 2 diabetes, regular consumption of beta-glucan-enriched oat bread did not improve long-term glycemic control relative to whole-grain wheat bread under real-world conditions [54]. In metabolically challenged adults following low-gluten diets, an oat-rich diet reduced LDL cholesterol more than a rice-rich diet, but the contrast also changed beta-glucan, fiber, and the wider food matrix [55]. These studies strengthen the food-level evidence base; the avenanthramide-specific question requires matched exposure contrasts.

The attribution problem is especially clear for lipid outcomes. A meta-analysis of 58 randomized trials found that a median 3.5 g/day dose of oat beta-glucan lowered LDL cholesterol, non-HDL cholesterol, and apolipoprotein B [4]. A separate meta-analysis of randomized trials likewise reported cholesterol lowering with oat beta-glucan and identified baseline LDL cholesterol and diabetes status as effect modifiers [56]. The physical properties of beta-glucan are also central to its physiological effects [38]. A cholesterol response after an oat food is not enough to implicate avenanthramides unless beta-glucan and matrix features are held constant and avenanthramide exposure is verified.

Among the studies identified and appraised in this narrative review, none has shown that a purified or analytically characterized avenanthramide intervention improves body weight, visceral adiposity, hepatic fat, insulin sensitivity, or hard cardiometabolic outcomes in people with obesity. Current evidence supports biological plausibility, measured exposure, and small stress-model effects; targeted efficacy trials are needed before clinical claims can be made.

Registry records checked on 7 September 2026 illustrate ongoing reporting gaps. NCT01527604 is listed as completed, with 16 adults with obesity and central adiposity enrolled in an eight-week comparison of avenanthramide-enriched oat muffins and refined-flour muffins; no results are posted [57]. NCT07371598 remains listed as recruiting, with an estimated enrollment of 38 and a crossover muffin comparison assessing glycemic responses; the registry records current status without outcome data and therefore does not inform recruitment completion or efficacy [58]. Registry access dates are separate from the review’s historical search and extraction dates.

Table 2 compares representative cell, animal, pharmacokinetic, and controlled food studies. Purified-compound experiments offer attribution within the tested model; pharmacokinetics establishes absorption and the timing and identity of circulating analytes. Enriched-cookie trials assess biomarkers under exercise stress, whereas whole-oat trials assess metabolic outcomes alongside changes in fiber and the food matrix. These designs support exposure and biological plausibility, and AVN-specific clinical benefit still requires direct testing.

**Table 2:** Representative avenanthramide and oat studies: designs, exposures, findings, and interpretive limits.

Study Design and System	Exposure/Intervention	Main Findings	Interpretation and Limitations
**Cell experiment** Fatty-acid-induced HepG2 cells [14]	AVNs A, B, and C; 100 μmol/L for the mechanistic experiments	Improved glucose handling and altered insulin-signaling, glycogen-synthesis, and gluconeogenic proteins.	Mechanistic evidence at a supraphysiologic concentration; not human efficacy.
**Animal intervention** High-fat-diet-fed mice [15]	Defined AVN-B during high-fat feeding	Reduced weight gain and improved hepatic and serum lipid indices, with fatty-acid-metabolism signals.	Compound-specific preclinical evidence; species and dose limit translation.
**Animal intervention** High-fat-diet-fed mice [16]	AVN-rich oat extract during high-fat feeding	Reduced weight gain, oxidative stress, and inflammatory markers, with altered gut microbial outcomes.	Preparation-level evidence; mixture attribution is weaker than for a purified congener.
**Pharmacokinetic and metabotype studies** Healthy adults [18,19,22]	Enriched mixture, AVN cookies, oat bran, and solid/liquid oat foods	Detected parent AVNs, conjugates, methylated products, and microbial metabolites; kinetics varied by food form and participant.	Establishes exposure, analytes, and sampling windows; not clinical efficacy.
**Randomized oat-food trial** 68 adults with metabolic syndrome [42]	Two-day high-dose oats versus a macronutrient-adapted oat-free diet; six-week moderate oats versus continuation of a Western diet	Short-term lipid changes accompanied phenolic and microbial shifts; the six-week intervention did not reduce lipids between groups.	Strong food-to-host evidence; causal attribution is not AVN-specific.
**Small double-blind food trials** 16 postmenopausal women, 16 young women, and 24 non-obese young adults [43,44,45]	Higher- versus lower-AVN oat cookies for eight weeks, with eccentric exercise before and after supplementation	Selected exercise-associated inflammatory biomarkers differed by treatment; CK, pain, or antioxidant-capacity changes were also shared across allocations. See Table S2.	Small stress-model studies; not obesity-efficacy trials. Small samples, incomplete effect uncertainty, and limited adverse-event reporting constrain interpretation.
**Randomized whole-oat trial** 40 randomized adults with BMI ≥ 27 kg/m^2^; 34 completers (16 oat, 18 control) [46]	Beta-glucan-containing oat cereal for 12 weeks	Anthropometric, cholesterol, and ALT changes favored oat cereal; ultrasound fatty-liver scores did not differ between groups. Vital signs and laboratory safety markers were assessed.	Food-level result; AVN-specific attribution is not possible without a matched contrast and exposure verification.
**Controlled oat-food trials** Adults at metabolic risk or with metabolic challenge [54,55]	Beta-glucan oat bread versus whole-grain wheat; oat-rich versus rice-rich diet	Oat bread did not improve long-term glycemic control; oat-rich versus rice-rich diets favored LDL cholesterol. Beta-glucan, fiber, and matrix components differed.	Strengthens food-level evidence, not an AVN-specific claim.

Abbreviations: ALT, alanine aminotransferase; AVN/AVNs, avenanthramide(s); AVN-B, avenanthramide B; BMI, body mass index; CK, creatine kinase; LDL, low-density lipoprotein; μmol/L, micromoles per liter.

### 7.4. Tolerability and Adverse Events

Safety information differs materially across intervention formats. In the 2014 enriched-cookie trial, measured glutathione/redox variables did not indicate major disruption of endogenous antioxidant defenses, but this limited biochemical assessment was not a systematic adverse-event evaluation [43]. The checked full article does not provide event counts or an explicit no-adverse-events statement. The 2020 trial used health-history and gastrointestinal-tolerability questionnaires to determine eligibility and excluded oat allergy and important disorders; it reports neither intervention-emergent adverse-event counts nor results of longitudinal tolerability monitoring [45]. Exercise-induced soreness and CK changes were challenge outcomes, not evidence of ingestion-related adverse reactions. The 2016 investigators reported no signs of adverse effects when discussing the measured redox and antioxidant responses; the full article describes no prospective adverse-event surveillance or event counts [44].

Whole-oat trials provide separate food-level tolerability evidence. In the 2025 beta-glucan-enriched bread trial, seven participants in the oat group and two controls withdrew because of adverse events such as stomach problems; no serious adverse events were reported [54]. The 2026 metabolic-syndrome trial reported no severe diet-related side effects and included kidney and liver tests and a blood count as safety parameters [42]. These observations concern the tested foods and durations. Safety of higher-dose extracts or purified avenanthramides requires separate monitoring. Table S2 distinguishes measured safety-related variables, explicit safety results, and unverified or unreported information.

The 12-week oat-cereal trial in Taiwan randomized 40 adults with BMI ≥ 27 kg/m^2^ and analyzed 34 completers (16 oat, 18 control) [46]. Its methods specify safety assessments at weeks 0, 6, and 12, including vital signs, renal and liver-related measures, and blood/urine tests. The investigators reported tolerability and no adverse effects, with no adverse change in the listed safety markers in the oat group. Six participants withdrew: one with hyperglycemia, one with elevated triglycerides, and four who missed follow-up; the report does not establish that the two metabolic abnormalities were caused by oat intake. This is an explicit safety statement for an oat food with selected monitoring; isolated avenanthramide safety requires separate evidence [46].

### 7.5. Criteria for Interpreting Human Evidence

A human study can support an avenanthramide-specific interpretation only when it documents the consumed food’s congener profile and stability, a comparator matched for beta-glucan and major matrix features, and an exposure measure showing that the planned contrast occurred in participants [18,19,22]. The endpoint should match the biological claim: lipid endpoints for a lipid hypothesis, validated liver-fat measurement for a steatosis hypothesis, and a direct or validated insulin-sensitivity measure for an insulin-resistance hypothesis. These features align with current recommendations for nutrition feeding trials [40].

Studies with unverified composition or exposure provide food-level evidence. A chemically defined preparation without a clinically relevant endpoint provides mechanistic or exposure evidence. This distinction keeps feasibility, biomarker, and efficacy claims separate.

## 8. Translational Research Priorities

The central translational question is specific: does a defined, nutritionally attainable increment in avenanthramide exposure improve a prespecified metabolic endpoint beyond the effects of beta-glucan and the wider oat matrix? A credible test has to control food chemistry, systemic exposure, biological context, and clinical outcome together [4,18,42].

### 8.1. Chemical Definition Must Precede Biological Interpretation

Intervention papers need batch-level quantification of avenanthramides A, B, and C rather than total avenanthramides alone. Reporting per gram of dry matter and per serving, together with the assay platform, analytical standards, limits of quantification, storage conditions, and stability through the intervention, would make studies easier to compare [7,31,35]. This matters because cultivar, processing, storage, and germination can alter composition, and equal totals can conceal different congener profiles [26,33,34]. Recent authentic-standard LC-MS/MS work further showed that calibration based on one avenanthramide structural type can underestimate other types in commercial oat products, which argues for reporting individual congeners rather than totals alone [30].

For food interventions, the same report also needs beta-glucan content, major phenolic acids, energy, macronutrients, particle size or physical form, and processing history of the comparator [7,31,37]. This information is needed to interpret an enriched-food benefit at the product level and to match the claim to the chemical composition and intervention contrast [40].

### 8.2. Dose Selection Guided by Measured Exposure

The administered milligram dose is an incomplete surrogate for biological exposure. Human studies demonstrate parent compounds and diverse host- and microbiota-derived metabolites after oat products, with product form and interindividual metabolism changing the concentration-time profile [18,19,22]. Dose-ranging work can establish repeated parent and metabolite exposure before efficacy testing and show whether the candidate dose is food-deliverable.

Mechanistic experiments are most informative when their concentration ranges are compared with measured free and conjugated circulating species [18,19,22]. Without that comparison, the experiment remains a supraphysiological mechanistic probe; it may still identify a targetable pathway. Parent-compound potency in a cell line is not a substitute for activity of the chemical species present after food consumption.

### 8.3. Matched Comparators and Endpoint Discipline in Causal Food Trials

A clean efficacy trial would randomize participants with a defined metabolic phenotype to high- and low-avenanthramide foods matched for beta-glucan, energy, macronutrients, sensory properties, and manufacturing history. The intended causal contrast is a defined avenanthramide increment within an otherwise matched diet. This point is especially important for lipid endpoints because beta-glucan has established LDL-cholesterol effects and can otherwise dominate the outcome [4,38].

Primary endpoints have to follow the hypothesis and target population. A lipid hypothesis calls for an a priori lipid endpoint; a liver-fat hypothesis calls for validated hepatic-fat measurement; and an insulin-resistance hypothesis calls for a direct or well-validated insulin-sensitivity outcome. Secondary inflammatory, redox, microbiome, and multi-omics measures strengthen interpretation, whereas clinical benefit depends on the prespecified primary endpoint. The recent whole-oat metabolic-syndrome study illustrates both the value and the attribution limit of this approach [42]. Table 3 summarizes the conclusions supported by each evidence tier and the attribution gaps that future trials need to address.

**Table 3:** Evidence-to-claim map for avenanthramide-specific and food-level metabolic effects.

Evidence Tier	Representative Design	AVN Attribution and Endpoint Relevance	Conclusion Supported
**Defined congener, animal**	Avenanthramide B in HFD-fed mice [15]	Defined AVN contrast; no human exposure data; obesity phenotype in mice	Compound-specific preclinical evidence only; cannot establish human dietary efficacy.
**Human pharmacokinetics**	Enriched mixture, oat cookies, oat bran, and solid/liquid oat foods [18,19,22]	Parent AVNs and metabolites measured in plasma or urine; no clinical endpoint	Defines analytes, kinetics, and sampling windows; does not establish efficacy.
**AVN-enriched food RCTs**	High- versus low-AVN cookies for 8 weeks; *n* = 16, 16, and 24 [43,44,45]	Food AVN contrast; no circulating or urinary AVN measurements reported in the three checked full reports; exercise-challenge outcomes	Supports a candidate effect on exercise-associated inflammatory responses, not obesity or metabolic-disease efficacy.
**Whole-oat RCT in obesity**	Beta-glucan-containing oat cereal for 12 weeks; 40 randomized, 34 completers [46]	AVN content and systemic exposure not reported; obesity-related outcomes assessed	Can support a food-level result; AVN-specific attribution is not possible.
**Whole-oat RCT in metabolic syndrome**	Two-day high-dose and six-week moderate oat diets; 68 participants across two parallel studies [42]	Oat-food contrast with phenolic and microbial profiling; AVNs not verified as the causal contrast; lipid outcomes	Strong food-to-host evidence; the candidate mediators are not AVN-specific.
**Recent controlled oat-food RCTs**	Beta-glucan oat bread versus whole-grain wheat; oat-rich versus rice-rich diets [54,55]	Food comparators and glycemic or lipid endpoints; the interventions were not designed to isolate an AVN contrast	Strengthens evidence for oat or beta-glucan foods, not for an AVN-specific effect.

Abbreviations: AVN, avenanthramide; HFD, high-fat diet; RCT, randomized controlled trial.

### 8.4. Metabotype as an Effect Modifier

Faecalibacterium prausnitzii and related microbial features provide a testable source of heterogeneity [24]. In the oat metabolic-syndrome trial, microbial and phenolic measurements were also related to cholesterol response at the food level [42]. These findings support prespecified effect-modification analyses of metabotype and treatment response.

### 8.5. Safety and Claim Boundaries for Responsible Translation

Tolerability findings for conventional oat foods cannot be used to presume safety or efficacy of a concentrated avenanthramide preparation. Higher-dose studies should document adverse events, laboratory safety measures, concomitant medication use, and intervention stability and impurity profiles [40]. Food-level cardiometabolic benefits can be discussed as matrix effects; prevention or treatment claims for avenanthramides themselves need compound-specific trials. NCT06101784, a placebo-controlled oral-AVN dose study, is listed as completed with 92 participants and planned adverse-event, laboratory, vital-sign, and electrocardiographic assessments. Because no results were posted on 7 September 2026, the planned monitoring describes study design rather than observed safety [59].

Clinical wording should follow demonstrated effects in the relevant population and endpoint. At present, avenanthramides are a measurable bioactive system with plausible mechanisms, and prevention or treatment claims require compound-specific clinical evidence.

### 8.6. Dietary Context and Population Generalizability

The geographic setting of a study informs external validity but does not determine who can biologically benefit. Direct enriched-cookie evidence includes US cohorts recruited in Madison and Minneapolis–Saint Paul [43,45]; other evidence includes a Swedish pharmacokinetic study, a Finnish oat-versus-rice trial, and a German metabolic-syndrome trial [22,42,55]. These selected populations and products do not demonstrate an avenanthramide-specific benefit across regions, and they do not support restricting a potential benefit to populations with longstanding oat consumption. In particular, botanical origin or the number of oat-consuming countries is not a substitute for comparative human efficacy evidence.

Dietary traditions, sensory preferences, cost, availability, and the foods displaced by oats can affect achievable intake and adherence independently of biological efficacy. Future trials should recruit metabolically relevant participants across dietary settings, record habitual oat and fiber intake, and use culturally acceptable foods with analytically matched comparators. Prespecified analyses of baseline diet, sex, age, metabolic phenotype, and measured metabolite exposure could test effect modification, while feasibility and tolerability should be evaluated in their own right [22,24,40].

Product suitability is especially relevant when oats are used in gluten-free diets. In a Canadian survey of 133 commercial oat samples, 88% exceeded 20 mg/kg gluten from other grains, with potential cross-contact across production and handling stages [60]. Trials involving celiac disease or non-celiac gluten sensitivity should report oat cultivar, purity or certification procedures, contamination testing, processing, and batch identity. Agricultural practices and pesticide residues require direct product-level measurement when relevant; neither can be inferred from geographic origin or avenanthramide concentration.

## 9. Conclusions

Avenanthramides are oat phenolic amides with measurable human exposure and reported actions in inflammatory, redox, hepatic glucose, lipid, intestinal, and vascular pathways. The evidence appraised in this narrative review establishes human exposure and biological plausibility, while compound-specific metabolic efficacy in humans still requires direct clinical evidence. The next useful studies are therefore practical as much as mechanistic: define and stabilize the preparation, verify parent and metabolite exposure, match beta-glucan and the oat matrix between groups, recruit metabolically relevant populations, and test a prespecified clinical endpoint. Avenanthramide-specific metabolic efficacy therefore requires direct clinical testing.

## Figures and Tables

**Table 1:** Chemical and food-matrix evidence needed to interpret avenanthramide exposure in metabolic research.

Section	Key Evidence	Exposure Interpretation	Implication for Metabolic Studies
3.1 Structure and nomenclature	A = 2p; B = 2f; C = 2c. Minor congeners expand the analytical profile [5,9,26].	Equal totals can represent different congener mixtures. Chemical antioxidant rankings do not establish in vivo efficacy [10].	Report individual congeners and identify the tested parent compounds and relevant metabolites.
3.2 Content and distribution	Oat foods, milling fractions, cultivars, and growing conditions differ in measured content [7,8,11]. Authentic standards reduce calibration bias [30].	A food category is not a dose. Concentrations depend on extraction, calibration, and dry-matter versus as-consumed reporting [7,8,27].	Analyze the final edible batches; report units, moisture basis, assay, congener panel, and amount per serving.
3.3 Processing and stability	Germination, heat, storage, and matrix structure affect composition, recovery, and release [26,31,33]. Food form changes human kinetics [22].	A measured change can reflect degradation or altered extractability. The same ingested dose can produce different exposure profiles.	Verify stability during consumption; match beta-glucan and major matrix features; measure parent and metabolite exposure.

The table does not pool concentrations across products or analytical methods. Abbreviations: AVN, avenanthramide; A, avenanthramide 2p; B, avenanthramide 2f; C, avenanthramide 2c.

## Data Availability

No new data were created or analyzed in this study. Data sharing is not applicable to this article.

## Supplementary Materials

The following supporting information can be downloaded at: https://www.mdpi.com/article/10.3390/nu18183066/s1, Table S1, Representative map of cited avenanthramide exposure, intervention, and registry studies [14,15,16,18,19,21,22,24,42,43,44,45,46,54,55,57,58,59,61]; and Table S2, Design, quantitative findings, and safety-reporting status of the three avenanthramide-enriched cookie trials, [42,43,44,45,54,55,61]. Reference numbers correspond to the revised main manuscript.
