# Supplementary material for: Avenanthramides in Obesity-Related Metabolic Dysfunction: Mechanisms, Evidence Attribution, and Translational Priorities
Source: Nutrients. 2026 Sep 19;18(18):3066. doi: 10.3390/nu18183066 (PMC13610561; doi:10.3390/nu18183066)
Supplement: Supplementary file 1 [file nutrients-18-03066-s001.zip › nutrients-4529161-supplementary.pdf]

Supplementary Methods and Tables S1 and S2. Representative evidence map and appraisal of avenanthramide-enriched food trials

Supplementary Methods. Narrative scope, source identification, and evidence charting

PubMed/MEDLINE was the primary biomedical source described for the historical search. Additional source checking used Crossref and publisher pages, Google Scholar and targeted web-title searches, ClinicalTrials.gov, and backward citation chaining from cited studies and recent reviews. Historical search and extraction cutoffs remain distinct from revision verification below. The initial search covered database inception through 7 July 2026; evidence extraction ended on 31 July 2026; and the final PubMed refresh was run on 7 August 2026.

PubMed query recorded in the source manuscript: ("avenanthramide*" OR "avenanthramide A" OR "avenanthramide B" OR "avenanthramide C" OR "avenanthramide 2p" OR "avenanthramide 2f" OR "avenanthramide 2c") AND ("oat" OR "oats" OR "Avena sativa") AND (absorption OR bioavailability OR metabol* OR microbiota OR inflammation OR oxidative OR obesity OR adipose OR insulin OR glucose OR lipid OR hepatic OR steatosis OR "clinical trial" OR intervention). This query records the biomedical search terms; supplementary source checking addresses food-chemistry, analytical-method, publisher-indexed, and trial-registration records that may fall outside PubMed indexing. The recorded core query did not separately enumerate extract, flour, bran, cookie, or whole-grain food forms; targeted revision checks paired those food-form terms with avenanthramide and oat terminology.

Narrative scope and selection rationale. Sources within scope report or review avenanthramide chemistry, occurrence, processing stability, digestion, absorption, host or microbial metabolism, tissue exposure, obesity-related mechanisms, human intervention evidence, or registered trial designs relevant to metabolic health. Reviews were used for background and cross-checking; original studies were prioritized for evidence attribution. Table S1 presents representative cited examples spanning cell and animal models, human exposure studies, enriched foods, whole-oat interventions, and trial registries. They illustrate intervention definition, exposure measurement, and design-specific inference; the table is not an exhaustive inventory. Xiaoyu Zhang and Hao Sun screened and selected the literature, extracted study information, and checked the extracted data. Only English-language literature was searched. Evidence extraction was completed through 31 July 2026.

Scope boundaries. Sources outside scope do not involve oats or avenanthramides, use avenanthramide terminology without chemical or biological relevance to the review question, address general oat processing without interpretable exposure or matrix information, or duplicate evidence covered by a fuller report. General plant-defense, agronomic, dermatologic, and unrelated supplement findings are not treated as evidence of obesity-related clinical efficacy.

Evidence charting and synthesis. Charted items include intervention format, chemical definition of the exposure, population or model, measured parent or metabolite exposure, metabolic or inflammatory readouts, and the highest inference supported by the design. Because the evidence base spans purified compounds, enriched foods, whole-oat foods, cell models, animal models, pharmacokinetic studies, and small human trials, findings were synthesized narratively rather than pooled statistically. Table S2 adds allocation, exercise protocols, reported numerical effects and uncertainty, and safety-reporting status for the three cookie trials. Values were taken from accessible original text or tables; graphs were not digitized and confidence intervals were not reconstructed. Information absent from a checked report is distinguished from information unverified because full text was inaccessible. The narrative design was selected to connect chemical identity, food processing, exposure biology, mechanisms, and clinical interpretation. Heterogeneity in populations, interventions, comparators, and outcomes precluded a meaningful pooled treatment-effect estimate; this does not preclude a future systematic review of a narrower clinical question.

Revision verification. On 7 September 2026, a targeted Europe PMC title/abstract check used TITLE_ABS:avenanthramide* AND FIRST_PDATE:[2025-01-01 TO 2026-09-07]; this returned 48 records. A broader exploratory query, avenanthramide* AND (human OR clinical OR trial OR metabolic OR obesity OR safety), retrieved many full-text/background matches and was used only for targeted source discovery. This was not a systematic screening update, and no PRISMA selection counts are inferred from these retrievals. The 2014 and 2020 enriched-cookie full reports and the 2016 publisher abstract and Tables 1–2 were checked on 7 September. The complete 2016 article, publicly uploaded by coauthor Yi-Fang Chu, was checked on 9 September 2026 (DOI: 10.1007/s00421-015-3244-3). ClinicalTrials.gov records NCT01527604, NCT07371598, NCT02584946 [61], and NCT06101784 were accessed separately on the same date; none contained posted results. Registry status and planned measurements are not trial results.

Table S1. Representative map of cited avenanthramide exposure, intervention, and registry studies.

Evidence tier	Ref.	Intervention or model	Population/system	Key readout	Highest defensible inference
Human pharmacokinetics	[18]	Acute enriched AVN mixture	Healthy older adults	Plasma free and conjugated AVN analytes at nanomolar concentrations	Demonstrates absorption and rapid host metabolism; does not establish metabolic efficacy.
Human pharmacokinetics	[19]	High- versus low-AVN oat cookies	16 healthy adults in an acute cookie pharmacokinetic study [19]	Parent-congener plasma time profiles	Food-level AVN contrast can produce measurable, congener-specific exposure differences.
Human pharmacokinetics	[21]	Oat bran consumption	Healthy adults	Plasma and urine AVN metabolite profiles	Supports the importance of host-conjugated metabolites in systemic exposure.
Human pharmacokinetics	[22]	Solid and liquid oat products, repeated intake	21 adults	Analyte kinetics and adherence-compatible exposure measures	Food form and repeated intake can alter exposure timing and should be measured, not assumed.
Human metabotype	[24]	Whole-grain oat intervention	Healthy adults	Early parent compounds, later microbial products, and microbial correlates	Supports heterogeneous host-microbial handling; a metabotype may modify exposure-response relations.

Evidence tier	Ref.	Intervention or model	Population/system	Key readout	Highest defensible inference
AVN-enriched food RCT	[43]	Two cookies/day; 9.2 versus 0.4 mg AVNs/day for 8 weeks [43]	16 postmenopausal women; 8 per group; age 50–80 years; mean baseline BMI 29.01 and 26.40 kg/m²	Exercise-challenge inflammatory/redox outcomes; product AVNs assayed, not circulating/urinary AVNs	Candidate biomarker response in a small exercise study; no weight/BMI change. Event counts not reported in the checked full text. See Table S2.
AVN-enriched food RCT	[44]	Two cookies/day; 9.2 versus 0.4 mg AVNs/day for 8 weeks [44]	16 women aged 18–30 years; 8 per group; mean baseline BMI 23.9 and 23.3 kg/m²	Exercise-challenge inflammatory/redox biomarkers; cookie AVNs assayed, no plasma/urinary AVNs reported	Full text checked; biomarker contrasts lack numerical effect estimates and 95% CIs. Limited safety statement without systematic event reporting. See Table S2.
AVN-enriched food RCT	[45]	Two cookies/day; 20.6 mg AVNs/day versus non-detectable AVNs (reported as 0 mg/day), for 8 weeks [45]	24 non-obese adults; 11 men/13 women; 12 per group; mean BMI 22.3 kg/m²	Inflammatory biomarkers and administered dietary AVN dose; no circulating or urinary AVN exposure measurement reported	Some treatment-related biomarker interactions; CK/pain improvements also occurred across groups. Dietary dose is not measured systemic exposure. See Table S2.
Whole-oat RCT	[46]	Oat intake intervention	40 adults with BMI ≥ 27 kg/m² randomized; 34 completers (16 oat, 18 control); Taiwan	Obesity-associated metabolic outcomes	Food-level metabolic findings; no between-group ultrasound fatty-liver improvement. Selected safety measures and an explicit no-adverse-effects statement; six withdrawals included two metabolic abnormalities without established oat causation.
Whole-oat RCT	[42]	Oat intervention	Adults with metabolic syndrome	Metabolic-syndrome related outcomes	Supports an oat-food hypothesis in a target population, not an isolated AVN effect.
Whole-oat food trial	[54]	Beta-glucan oat bread versus whole-grain wheat bread	Adults at risk of type 2 diabetes	Long-term glycaemic-control outcomes	A beta-glucan oat-food contrast did not improve real-world glycaemic control; food-matrix and fiber effects remain relevant to interpretation.
Whole-oat food trial	[55]	Oat-rich versus rice-rich low-gluten diet	Adults with a metabolic challenge	LDL-cholesterol and other cardiometabolic outcomes	A favorable food-level LDL contrast cannot by itself distinguish AVN effects from beta-glucan, fiber, or matrix effects.
Animal study	[15]	Defined AVN-B administration in high-fat-diet model	Mice	Hepatic inflammation, oxidative stress, and lipid-related outcomes	Supports biological plausibility under a defined-congener exposure; dose and species limit direct human inference.
Animal study	[16]	AVN-rich preparation in high-fat-diet model	Mice	Adiposity and metabolic-inflammatory outcomes	Supports a preparation-level mechanistic hypothesis; mixture composition and dose need explicit reporting.
Cell study	[14]	Parent AVNs A, B, and C; 100 µmol/L in the mechanistic assays [14]	Free-fatty-acid-treated HepG2 cells	Glucose consumption, glycogen content, insulin-signaling and gluconeogenic proteins	Identifies cellular responses at a supraphysiologic experimental concentration; it does not prove a human tissue effect.
Registered human trial	[57]	Two AVN-enriched oat muffins/day versus refined-flour muffins for 56 days [57]	16 adults with obesity and central adiposity; completed registry record	Planned inflammatory, glucoregulatory, lipid, and blood-pressure outcomes	No posted results on 7 September 2026; registry design and completion status do not establish efficacy or safety.
Registered human trial	[58]	Controlled oat-muffin crossover	Estimated n = 38; crossover muffin trial; registry listed as recruiting [58]	Planned glycaemic and food-response outcomes	No posted results on 7 September 2026. The registry status may lag actual trial activity; no efficacy inference is supported.
Registered dose/safety study	[59]	Single and repeated oral AVN tablets versus placebo	92 participants; completed AvenActive record, Montreal, Canada	Planned adverse-event, laboratory, vital-sign, ECG, and pharmacokinetic outcomes	No posted results on 7 September 2026; planned monitoring is not demonstrated safety.

Reference numbers correspond to the revised main manuscript. The final column states the inference supported by the design, not a clinical-efficacy rating or a claim of exhaustive study identification. Table S1 abbreviations: Ref., reference; AVN/AVNs, avenanthramide(s); AVN-B, avenanthramide B; BMI, body mass index; CI, confidence interval; CK, creatine kinase; ECG, electrocardiogram; LDL, low-density lipoprotein; RCT, randomized controlled trial; NCT, ClinicalTrials.gov identifier; n, number of participants.

Table S2. Design, quantitative findings, and safety-reporting status of the three avenanthramide-enriched cookie trials.

Study and population	Dose, comparator, and challenge	Reported effects and uncertainty	Exposure and safety appraisal
Koenig et al., 2014 [43]<br>Madison, Wisconsin, USA; 16 postmenopausal women, age 50–80 years; 8 per arm. Baseline BMI: 29.01 ± 1.27 versus 26.40 ± 1.59 kg/m² (mean ± SEM, high-dose/control).	Two cookies daily: 9.2 versus 0.4 mg AVNs/day for 8 weeks. Cookie AVNs measured by HPLC.<br>Before and after supplementation: 4 × 15 min downhill walking, −9%, 4.0 km/h; 5-min rests. Samples: rest, 24 and 48 h.	Investigators described IL-1β as nearly 50% lower with high-dose cookies at rest and 24 h after the post-intervention challenge; not different at 48 h. No numerical between-group effect with 95% CI for this contrast. Figures use mean ± SEM.<br>Weight/BMI unchanged; CK did not differ by group; total antioxidant capacity increased in both groups. Methods specify $P < 0.00455$ for 11 comparisons; captions often use $P < 0.05$.	Full text checked (Methods; Results; Tables 1–2; Figures 1–6). Product dose was measured; plasma/urinary AVNs were not reported.<br>Redox/GSH variables were measured and interpreted by the investigators as not showing major antioxidant-system disruption. No adverse-event counts, systematic ascertainment method, or explicit no-events statement was identified. This is limited biochemical evidence, not demonstrated comprehensive safety.
Koenig et al., 2016 [44]<br>Madison, Wisconsin, USA; 16 women, age 18–30 years; 8 per arm. Baseline BMI: 23.9 ± 0.89 versus 23.3 ± 0.72 kg/m² (mean ± SEM). Diabetes, cardiovascular disease, and high-level training were excluded.	Two cookies daily: 9.2 versus 0.4 mg AVNs/day for 8 weeks; cookie AVNs verified by HPLC. Double-blind allocation.<br>Before and after supplementation: 4 × 15 min downhill running, −9%, 75% predicted maximal heart rate (220 − age); 5-min rests. Samples: rest, immediately after, and 24 h.	Publisher Table 2: resting GSH after intervention 5.88 ± 0.08 versus 5.38 ± 0.09 μmol/L; baseline 5.53 ± 0.09 versus 5.52 ± 0.09 (mean ± SEM; post-intervention between-group $P < 0.05$). No treatment-difference CI in that table.<br>IL-6 was lower immediately/24 h after exercise and NF-κB at 24 h with high-dose cookies ($P < 0.05$). Inflammatory figures show mean ± SEM/P values, without numerical between-group effects or 95% CIs. CK/TNF-α improved in both arms; weight/BMI unchanged. CK Figure 1: $n = 6$; other figures: $n = 8$; smaller CK sample unexplained.	Full text checked (Methods; Results; Tables 1–2; Figures 1–7; Discussion). Cookie dose measured; plasma/urinary AVNs not reported.<br>The investigators reported no signs of adverse effects in their discussion of redox and antioxidant responses. Prospective adverse-event surveillance and event counts are not described. The statement supports limited biochemical tolerability; chronic higher-dose effects remain uncertain.
Zhang et al., 2020 [45]<br>Minneapolis–Saint Paul, USA; 24 non-obese adults, 11 men/13 women; 12 per arm. Reported age 23.0 ± 1.2 years, BMI 22.3 ± 0.8 kg/m². Regular exercisers and specified disorders excluded.	Two cookies daily: 20.6 mg AVNs/day versus non-detectable AVNs, reported as 0 mg/day; 8 weeks. Initial exercise test, 8-week washout, then 8-week supplementation.<br>4 × 15 min downhill running, −10%, 75% maximal heart rate; 5-min rests. Samples: rest, 0, 4, 24, 48, 72 h.	CK 19.5%/23.6% lower at 24/48 h POST versus PRE across allocations; not an AVN effect. Pain also improved across arms.<br>Reported interactions: NRB/sVCAM-1 time × treatment; G-CSF supplementation × treatment; IL-1Ra three-way ($P < 0.05$). IL-6 time × treatment $P = 0.082$. No numerical effect estimates with 95% CIs for these contrasts; figures show mean ± SEM.	Full text checked (Methods; Results; Figures 2–9). Dietary dose reported; plasma/urinary AVNs not measured in the report.<br>Health-history/GI-tolerability questionnaires used for eligibility; oat allergy excluded. No intervention-emergent event counts or longitudinal tolerability results reported. Exercise soreness/CK are challenge outcomes. Registry NCT02584946 has no posted results.

Table S2 abbreviations: AVN(s), avenanthramide(s); BMI, body mass index; CI, confidence interval; CK, creatine kinase; G-CSF, granulocyte colony-stimulating factor; GI, gastrointestinal; GSH, reduced glutathione; HPLC, high-performance liquid chromatography; IL, interleukin; IL-1β/IL-6/IL-1Ra, interleukin-1 beta/interleukin-6/interleukin-1 receptor antagonist; NF-κB, nuclear factor kappa B; NRB, neutrophil respiratory burst; P, P value; PRE/POST, before/after supplementation; SEM, standard error of the mean; sVCAM-1, soluble vascular cell adhesion molecule-1; TNF-α, tumor necrosis factor alpha; USA, United States; NCT, ClinicalTrials.gov identifier; n, number of participants/observations.

Interpretation of Table S2. Values are the original reports' summaries, not recalculated effect estimates. SEM describes precision of an arm mean and is not SD or a confidence interval for a randomized contrast. No values were digitized from graphs. Small samples, multiple endpoints and time points, and repeated exercise challenges limit inference; differences in significance within separate arms do not themselves establish a between-group effect. The 2014 and 2020 full texts contained no linked supplementary safety results in the versions checked. The 2016 appraisal uses the complete author-uploaded article; no separate supplementary file was available for verification.

Separate food-level safety findings. Hjorth et al. [54] reported withdrawals attributed to adverse events, including stomach problems, in seven oat-bread participants and two controls, and no serious adverse events. Klümpen et al. [42] reported no severe diet-related side effects and monitored kidney/liver parameters and blood count. Mannila et al. [55] described adverse-effect contact with researchers and physician consultation; one participant was excluded for study-product intolerance and another for acute gastroenteritis. These are findings for multicomponent diets, not estimates of the safety of purified AVNs. The intolerance passage does not identify an AVN-related cause.
